# Risk-conscious correction of batch effects: maximising information extraction from high-throughput genomic datasets

**DOI:** 10.1186/s12859-016-1212-5

**Published:** 2016-09-01

**Authors:** Yalchin Oytam, Fariborz Sobhanmanesh, Konsta Duesing, Joshua C. Bowden, Megan Osmond-McLeod, Jason Ross

**Affiliations:** 1 CSIRO, Genomics and Cellular Sciences, Transformational Biology CP, North Ryde, NSW Australia; 2 CSIRO, Genomics and Cellular Sciences, Advanced Materials CP (Nanosafety), 11 Julius Avenue, North Ryde, NSW 2113 Australia; 3 CSIRO, IM&T, Science Applications, St Lucia, QLD Australia

**Keywords:** Batch effects, ComBat, High-throughput genomic data, Measurement noise, Principal component analysis, Singular value decomposition, Guided PCA

## Abstract

**Background:**

Batch effects are a persistent and pervasive form of measurement noise which undermine the scientific utility of high-throughput genomic datasets. At their most benign, they reduce the power of statistical tests resulting in actual effects going unidentified. At their worst, they constitute confounds and render datasets useless. Attempting to remove batch effects will result in some of the biologically meaningful component of the measurement (i.e. signal) being lost. We present and benchmark a novel technique, called *Harman*. Harman maximises the removal of batch noise with the constraint that the risk of also losing biologically meaningful component of the measurement is kept to a fraction which is set by the user.

**Results:**

Analyses of three independent publically available datasets reveal that Harman removes more batch noise and preserves more signal at the same time, than the current leading technique. Results also show that Harman is able to identify and remove batch effects no matter what their relative size compared to other sources of variation in the dataset. Of particular advantage for meta-analyses and data integration is Harman’s superior consistency in achieving comparable noise suppression - signal preservation trade-offs across multiple datasets, with differing number of treatments, replicates and processing batches.

**Conclusion:**

Harman’s ability to better remove batch noise, and better preserve biologically meaningful signal simultaneously within a single study, and maintain the user-set trade-off between batch noise rejection and signal preservation across different studies makes it an effective alternative method to deal with batch effects in high-throughput genomic datasets. Harman is flexible in terms of the data types it can process. It is available publically as an R package (https://bioconductor.org/packages/release/bioc/html/Harman.html), as well as a compiled Matlab package (http://www.bioinformatics.csiro.au/harman/) which does not require a Matlab license to run.

**Electronic supplementary material:**

The online version of this article (doi:10.1186/s12859-016-1212-5) contains supplementary material, which is available to authorized users.

## Background

Modern high-throughput genomic datasets are exquisite in their detail. The comprehensive range of measurements contained therein not only ameliorates, at least to a degree, reliance on narrow and specific *a priori* hypotheses, but also makes possible an appreciation of genetic behaviour at its fullest – i.e. at the level of interconnected gene networks. In this sense, modern genomics opens the door to forms of biological knowledge and thinking which would be difficult to attain with traditional methods of experimental biology.

There are challenges to be met in going from the rich detail in the datasets to a systemic understanding of genes. In our view, these can be separated into two main stages – first the establishment of reliable units of evidence, second the discovery of what these might mean at a global, systemic level. To illustrate via an example, suppose a genome-wide gene expression dataset resulting from an experiment comparing cellular response to a particular treatment against a control group. The first stage is to establish a reliable and exhaustive list of genes that are differentially expressed under the two conditions. The second is to go from the list of individual genes to a functional understanding of gene pathways, activated as a result of the treatment. *Batch effects*, the topic of this manuscript, belong to the first stage of challenges. They are a pervasive form of technical noise, which compromise individual measurements to varying degrees, and affects significantly the ability of analytical means used to identify those that vary between experimental conditions. Batch effects are found in gene expression microarray [1], sequencing [2], DNA methylation (e.g. [2–4]), copy number variation (e.g. [2, 5, 6]) and proteomic (e.g. [7]) datasets.

### Batch effects are structured patterns of distortion

High-throughput technologies in biology typically require a sequence of delicate and labour intensive procedures, involving a combination of reagents and specialist machinery, conducted under strictly controlled conditions. Frequently, the volume and nature of the work means that the laboratory process is broken into ‘batches’ – each batch consisting of a certain number of replicates to process – performed over a number of days. Batch effects consist of a series of structured patterns of measurement noise each of which permeates all replicates in a given processing batch, and which vary markedly from batch to batch. We describe batch distortion as being *structured*, because it has a spatial character – in the case of microarrays for example, it imprints upon the expression values of probesets depending on the location of their constituent probes [1]. A large number of probesets can have their values altered significantly by this kind of distortion, without it being reflected in measures that are not spatially sensitive. To illustrate the point, it is possible to distort the expression value of all probesets completely (by misallocating them the value of their preceding probeset) without at all changing, say, the quantile distribution of probeset values. As such, quantile normalisation techniques such as RMA [8] would be of limited use in correcting batch effects [2, 9, 10]. A helpful visual metaphor may be to think of a dried watermark, formed by an unintended splash of brush water on a fresh painting. Or rather, a printing machine with a software virus, which makes prints of paintings, produces a certain number of copies at a time, each set with the same ‘watermark’, and that watermark changes randomly from set to set. These ‘watermarks’ cannot be removed from a digital poster, simply by adjusting its mean or quantile intensities of red, green and blue. They can be altered, along with the unaffected parts of the painting and hence causing a ‘smearing’ effect, but not removed.

### Result of a stochastic interaction of process variables?

Batches being processed in different laboratories, by different personnel, subtle ambient differences (in temperature or humidity) in the same laboratory from one processing day to the next, and changes in reagents have been suggested and explored as the cause of batch effects [1, 2]. Evidence suggests batch effects are pervasive and persistent under best practice. Indeed, in the studies we conducted [11, 12], all the above mentioned factors were well controlled – the same laboratory (with controlled temperature and humidity), the same operator, and the same re-agents. Yet the data revealed significant batch effects, accounting for as high as 40 % of the variance in the data. Leek et al. [2] make the insightful observation that structured measurement noise such as batch effects are in fact not unique to high-dimensional genomic datasets (e.g. microarray or RNA-seq), or other types of high-dimensional data (e.g., mass spectroscopy), but also affect traditional ‘low-dimensional’ data where just a few measurements are involved. The distinction, they propose, is that batch effects are identifiable in high-dimensional datasets, but not so in traditional datasets and as such go unnoticed. If so, it may be useful to think of batch effects as stemming from a stochastic combination of many of the factors at play during laboratory processing of data capture equipment, which is not readily controllable or avoidable. A more achievable way of managing batch noise may be to dissociate it from the genuine biological signal component of the dataset, and remove it in an effective manner.

### Batch effects have a detrimental effect on the utility of datasets

In terms of scientific inference, batch effects are most problematic when they are aligned (i.e. strongly correlated) with treatment effects. Table 1A depicts one such example, an extreme yet not uncommon one, where each processing batch contains one type of treatment or experimental condition. The difference between a pair of treatments will be completely confounded by the typically larger difference between the two distinct patterns of batch distortion. An entire group of genes, invariant across the two experimental conditions yet with probesets altered differentially by batch distortion will appear to be differentially expressed [2, 13]. Moreover, these false positives may dominate those genes that are differentially expressed across the two experimental conditions, because they are likely to appear to have a larger difference in their expression levels. The common practice of selecting top differentially expressed genes for further analysis and exploration, as ranked by magnitude, may further exacerbate this problem – resulting in the exclusion of differentially expressed genes, in favour of false positives.

**Table 1:** Separating samples into processing batches

Batch 1	Batch 2	Batch 3	Batch 4
A			
T_1r1_ + B_1_	T_2r1_ + B_2_	T_3r1_ + B_3_	T_4r1_ + B_4_
T_1r2_ + B_1_	T_2r2_ + B_2_	T_3r2_ + B_3_	T_4r2_ + B_4_
T_1r3_ + B_1_	T_2r3_ + B_1_	T_3r3_ + B_3_	T_4r3_ + B_4_
T_1r4_ + B_1_	T_2r4_ + B_2_	T_3r4_ + B_3_	T_4r4_ + B_4_
B			
T_1r1_ + B_1_	T_1r2_ + B_2_	T_1r3_ + B_3_	T_1r4_ + B_4_
T_2r1_ + B_1_	T_2r2_ + B_2_	T_2r3_ + B_3_	T_2r4_ + B_4_
T_3r1_ + B_1_	T_3r2_ + B_1_	T_3r3_ + B_3_	T_3r4_ + B_4_
T_4r1_ + B_1_	T_4r2_ + B_2_	T_4r3_ + B_3_	T_4r4_ + B_4_

B denotes batch effects, T is treatment and the subscript r is the replicate of that treatment. (A): In this design, each batch consists of one type of treatment. Batch and treatments effects are completely confounded. When we attempt to measure the difference between two treatments, say T_1 _and T_2_, what we are actually measuring is (T_1_-T_2_) + (B_1_-B_2_). Moreover, (B_1_-B_2_) is typically likely to be much larger than (T_1_-T_2_). (B): This represents the optimal experimental design strategy, where all treatments are distributed equally across all batches. There is no confounding here, but differences between B_1_, B_2_, B_3_ and B_4_ artificially inflate within-treatment differences, and reduce the power of subsequent statistical tests

It is possible to avoid this issue by making batch and treatment effects orthogonal to one another via modified experimental and procedural design. Table 1B depicts the optimal case, where the replicates of each and every treatment are distributed equally across the batches, avoiding any confounding between batch and treatment effects. The closer we come to this ideal design, the less the confounding effect. However, even with ideal experimental design and no confounding of batch and treatment effects, there remains a fundamental problem. Differences between individual batch effects, B_n_ in Table 1B, will inflate within-treatment variances, diminishing the power of any between-treatment comparison tests. As a result genes that are actually differentially expressed between two experimental conditions will have their *p*-values elevated and will appear to be not differentially expressed (see also [2], p.736). Moreover, different probesets on a particular array are affected differently by batch effects, meaning that some genes will have their *p*-values altered a lot, some less so, and some not at all. This will distort the ranking of genes based on their *p*-value, also distorting the results of rank based false discovery correction methods such as Benjamini-Hochberg ([14]; see also [13], pp. 9–10).

The ideal solution to batch effects is to completely dissociate batch noise from genuine biological signal in the dataset, remove all of batch noise and none of the biological signal. In practice, however, removing noise carries with it the risk of also removing biological signal. One fundamental reason for this is that the distinction between signal and noise components, if attainable, is likely to be probabilistic rather than absolute. If genuine biological variance is removed along with batch noise, within-group variances are then artificially deflated making genes that are not differentially expressed appear as though they are. If we had multiple batch correction methods to choose from, the score by which we measure their effectiveness would have two dimensions – how much of the batch noise they remove, and how much of the biological signal they preserve.

### Outline

In this paper we describe a novel method which dissociates and removes the batch noise component in a dataset, with the constraint that the associated risk of also removing genuine biological signal is quantified and kept to a fraction set by the end user. If we set our confidence limit to .95, this would mean that the probability of some of what we remove not being batch effect but a feature of genuine biological signal is .05. The method works by first separating the data into its principal components. It scans each principal component for variance arising out of batch noise – as manifest by clustering of scores belonging to the same batch – and removes any that is found up to a point where the risk of removing biological signal is no more than the tolerance level set by the user. As the principal components collectively explain all the variance to be found in the dataset, scanning and if necessary correcting each of them means that batch effects are found and corrected, irrespective of how big or small they may be with respect to other factors accounting for the data variance. The principal components after removal of batch noise are recombined and transformed back into the original dataset format, ready to be used for any downstream analysis tailored for the initial dataset, without necessitating any additional data processing. We call this new method *Harman*, meaning (in Turkish and Persian) threshing yard where grain was separated from chaff in the days before Industrialisation. Harman has a precedent in and can be seen as a refinement of the work of Alter and colleagues [15, 16], who transformed genome-wide expression data into principal components, and then removed some of them entirely which they inferred to be dominated by batch effects.

ComBat [17] is a popular batch removal method, which has been shown to have the best overall performance in a recent comparative study [9] of six approaches including [18–21]. As such it makes for a good standard against which to compare any novel batch removal method. We compare the performance of Harman with that of ComBat in the context of three distinct, publically available genome-wide gene expression datasets. Two of these – an in vitro [11] and an in vivo [12] study – were generated in our laboratory. The third is the in vitro dataset used in ComBat’s development [17]. While all three are microarray datasets, it is important to note that both ComBat and Harman would be applicable in correcting RNA-seq datasets (e.g., [22]). We also use Harman regularly to correct large methylation datasets.

The performance measures used in the study are the removal of (batch) noise, and preservation of (biological) signal. For the sake of objectivity, and in the absence of knowing categorically what is signal and what is noise, we use a third party batch noise quantification to evaluate the two methods, the “guided-PCA” statistic developed by Reese et al., [23] (see Additional file 1 for further discussion). Guided-PCA *p*-values can be used as a measure of the probability of batch effects being present in the dataset. As *p*-values are a continuous rather than discrete score, they provide a continuum against which the batch noise suppression of different methods or trade-off settings can be measured. The (inversely) proportional relationship between g-PCA *p*-value and the magnitude of the batch effect as measured by g-PCA is further demonstrated in the Additional file 1. We compute this for each of the three datasets before correction, and after correction by the two methods. Against this metric, we measure what proportion of the raw data variance is preserved in the corrected datasets. A two-dimensional plot of the probability of batch effect existence and proportion of preserved variance post correction depicts the relative merit of the two batch effect removal methods (see Additional file 1 for a more detailed discussion).

## Results

**Fig. 1:**
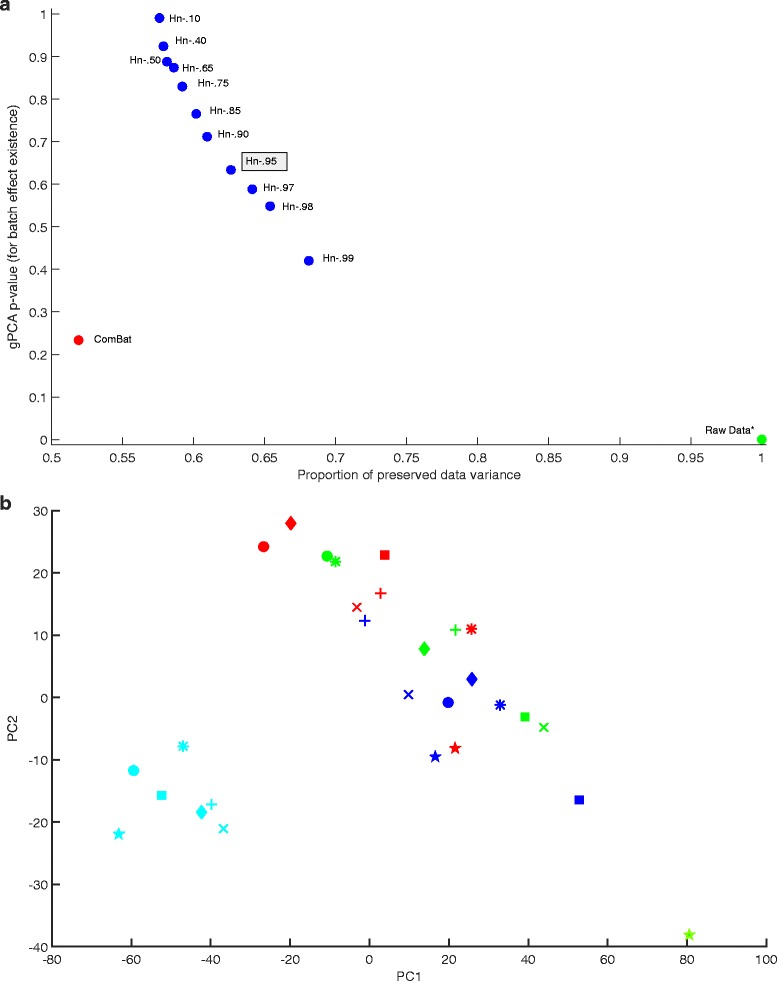
**a** gPCA *p*-value vs preserved data variance plot for Dataset 1 (Osmond-McLeod, Osmond et al., 2013), showing the scores for data before correction (*gPCA = .008), and after correction by ComBat and Harman batch effect removal methods. For Harman, the fractions in the labels denote the adjustable confidence threshold (=1-probability of overcorrection) for batch noise removal. Hn-.95 is highlighted as it may be the setting of choice for a typical dataset. On the vertical, the larger the *p*-value the lower the probability of batch noise presence as detected by gPCA (Reese et al, 2013). Raw data *p*-value of .008, indicates a prevalent batch noise component in the uncorrected dataset. The figure shows that ComBat falls below the Harman curve, indicating Harman’s superiority in terms of removing batch noise and preserving biological signal in the dataset. **b** First and second PCs for Dataset 1 (Osmond-McLeod, Osmond et al., 2013) before correction. The four colours represent the four processing batches. The shapes represent seven distinct treatments. The clustering of batches indicate the presence of batch effects in the first and second PCs of the data

Figure 1a above shows the batch correction results for Dataset 1, and Fig. 1b shows the PC plot for the first and second components. With a gPCA *p*-value of .008, the uncorrected dataset has a prevalent batch noise component, also evident in the PC plot. Consistent with this, the most conservative Harman setting with a confidence limit of .99 – which means correction stops when there is just 1 % chance that what is being removed may not be due to batch effects alone – results in a 32 % reduction in data variance. After correction by either method, *p*-value increases significantly suggesting the methods are capable of removing batch noise. The figure also reveals how the confidence limit for Harman operates as a trade-off coefficient between noise rejection and signal preservation. As the threshold is decreased, noise rejection increases as reflected by the gPCA *p*-value, and data variance decreases. The resulting Harman points can be thought of as constituting a performance curve for the correction method – one can choose to be at different points on the curve depending on the trade-off coefficient, but nevetheless is constrained to be on the curve. The ComBat point on the graph is below this curve.

**Fig. 2:**
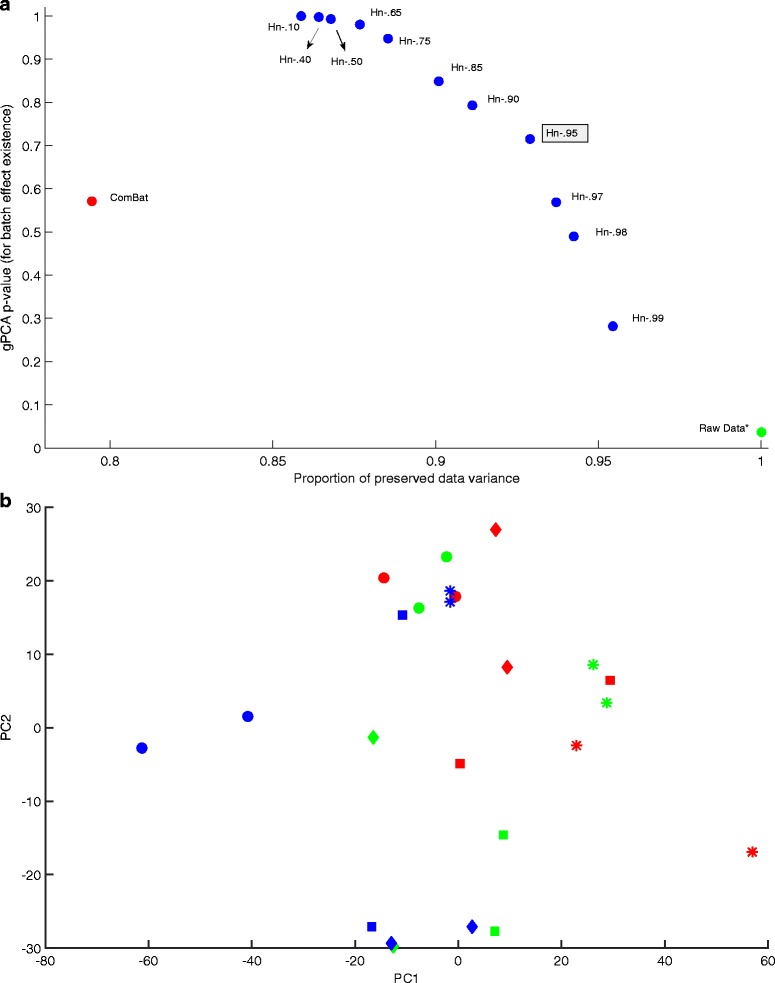
**a** gPCA *p*-value vs preserved data variance plot for Dataset 2 (Osmond-McLeod, Oytam et al., 2013), showing the scores for data before correction (*gPCA = .037), and after correction by ComBat and Harman batch effect removal methods. For Harman, the fractions in the labels denote the adjustable confidence threshold (=1-probability of overcorrection) for batch noise removal. Hn-.95 is highlighted as it may be the setting of choice for a typical dataset. On the vertical axis, the larger the *p*-value the lower the probability of batch noise presence as detected by gPCA (Reese et al, 2013). Raw data *p*-value of .037, indicates a batch noise component in the uncorrected dataset. The figure shows that ComBat falls below the Harman curve, indicating Harman’s superiority in terms of removing batch noise and preserving biological signal in the dataset. **b** First and second PCs for Dataset 2 (Osmond-McLeod, Oytam et al., 2013) before correction. The three colours represent the three processing batches. The shapes represent four distinct treatments. The clustering of batches (less pronounced than Dataset 1) indicate the presence of batch effects in the first and second PCs of the data

Dataset 2 results are depicted in Fig. 2. At .037, the gPCA *p*-value for the uncorrected dataset is small enough to indicate the presence of batch effects, if not to the same extent as in Dataset 1. Once again, this is consistent with the PC plot. Figure 2b indicates a batch effect but not to the same extent as Fig. 1b. Accordingly, both batch effect correction methods result in higher proportions of preserved data variance when compared to Dataset 1. As with Dataset 1 the gPCA *p*-value increases significantly after correction by either method. For Harman the confidence limit has the same trade-off characteristic between noise rejection and data variance preservation. The ComBat point falls below the Harman curve.

**Fig. 3:**
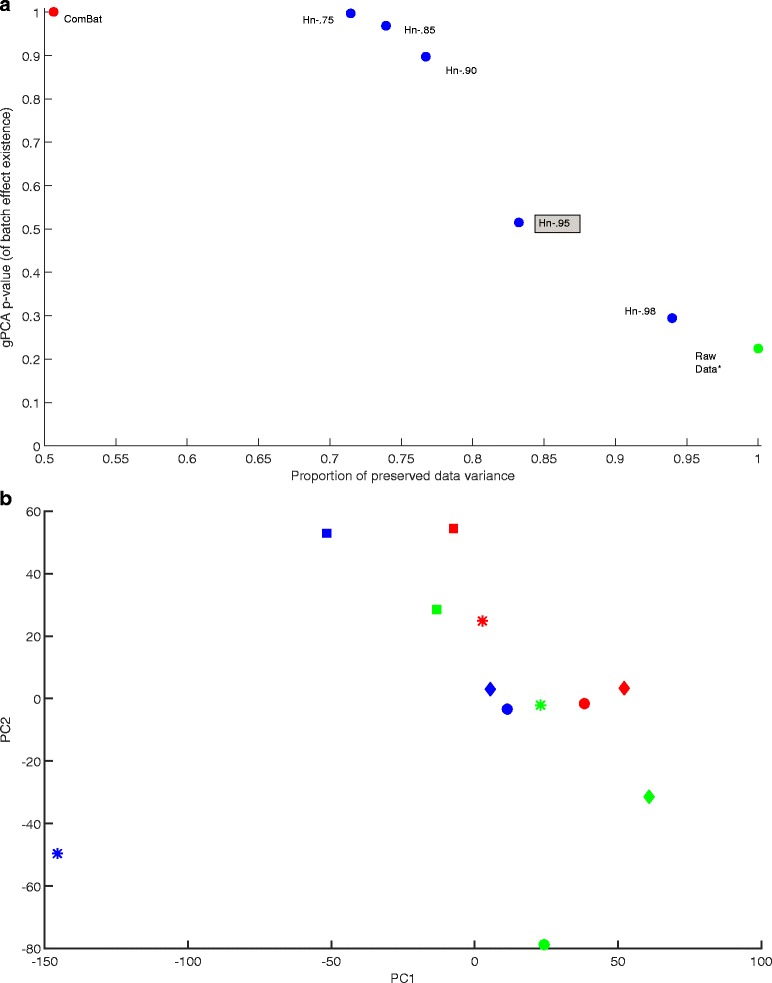
**a** gPCA *p*-value vs preserved data variance plot for Dataset 3 (Johnson et al., 2007), showing the scores for data before correction (*gPCA = .225), and after correction by ComBat and Harman batch effect removal methods. For Harman, the fractions in the labels denote the adjustable confidence threshold (=1-probability of overcorrection) for batch noise removal. Hn-.95 is highlighted as it may be the setting of choice for a typical dataset. On the vertical axis, the larger the *p*-value the lower the probability of batch noise presence as detected by gPCA (Reese et al, 2013). Raw data *p*-value of .225, indicates that the batch noise component in Dataset 3 is not as predominant as Datasets 1 and 2. The worst case scenario for both methods is that there is no batch effect in the dataset and what they do remove is genuine biological signal. ComBat removes 49 % (with gPCA *p*-value = 1) of the data variance, which is about the same proportion it removed Dataset 1 (48 %; gPCA *p*-value = .233), which had the most prevalent batch effect (gPCA *p*-value = .008). Harman (Hn.95) removes 17 % (gPCA *p*-value = .52), when it removed 37 % (gPCA *p*-value = .63) from Dataset 1. Hn-75 matches ComBat’s gPCA *p*-value of 1 while removing 20 percentage points less data variance. **b** A plot of first and second PCs for Dataset 3 before correction (Johnson et al., 2007). The three colours represent the three processing batches. The shapes represent four distinct experimental conditions. The figure indicates that within-treatment variability is a larger source of data variance than batch effects in the top two principal components

Dataset 3 shows (Fig. 3a), as with Datasets 1 and 2, that gPCA *p*-value increases after correction by ComBat or Harman, and that for the latter the confidence limit sets the trade-off between noise rejection and data variance preservation. It also produces some distinct results. The gPCA *p*-value for the uncorrected data is .225, which indicates that there is much less batch noise in Data 3 than in the other two datasets, if any at all. Indeed, Fig. 3b indicates that treatment variability (in particular, in the treatment group denoted by “*”) is a larger source of data variance than batch effects in the first two principal components. Harman (.95) removes 17 % (gPCA *p*-value = .52), compared to the 37 % (gPCA *p*-value = .63) it removed from Dataset 1. ComBat removes 49 % (with gPCA *p*-value = 1) of the data variance, about the same proportion it removed from Dataset 1 (48 %; gPCA *p*-value = .233) which has the most prevalent batch effect of all datasets (gPCA *p*-value = .008). Furthermore, Harman (.75) matches ComBat’s gPCA *p*-value of 1 while removing 20 percentage points less data variance.

Given the unexpectedly high *p*-value for the raw data, it is worth exploring further. With Harman, it is possible to dissociate the principal components in which it finds and removes batch effects, and whether these are in any way different for Dataset 3 than the other datasets. Table 2A below shows the amount of batch correction applied to the first 8 principle components for the three datasets. A score of 1 means there is no correction. The closer the number to 0, the bigger the correction. The remaining principal components not included in the dataset show no or negligible batch correction. Table 2B shows the proportion of overall data variance explained by each principal component.

**Table 2:** The varying nature of batch effects in the three datasets as detected by Harman

PC indices	1	2	3	4	5	6	7	8
A. Correction Vector (Hn-.95)								
Dataset 1	0.26	0.33	0.51	0.9	0.44	0.85	0.74	1
Dataset 2	0.42	1	0.93	1	0.99	1	1	0.95
Dataset 3	0.76	1	0.35	0.69	1	1	1	1
B. % of data variance explained by PC								
Dataset 1	43.4 %	9.5 %	4.8 %	4.3 %	2.7 %	2.4 %	2.2 %	2.0 %
Dataset 2	19.1 %	11.5 %	6.9 %	4.6 %	4.3 %	4.0 %	3.6 %	3.6 %
Dataset 3	33.9 %	17.2 %	16.0 %	8.6 %	5.8 %	4.5 %	3.7 %	3.3 %

(A) Shows the ‘correction vector’ spanning the first eight principal components for the three datasets resulting from Harman (.95). No or negligible correction were detected for the remaining PCs. A score of 1 means no correction, whereas a score of 0 means maximum correction within the confines of Harman. (B) Shows the relative proportion of overall variance explained by each of the (first eight PCs) for the three datasets

**Fig. 4:**
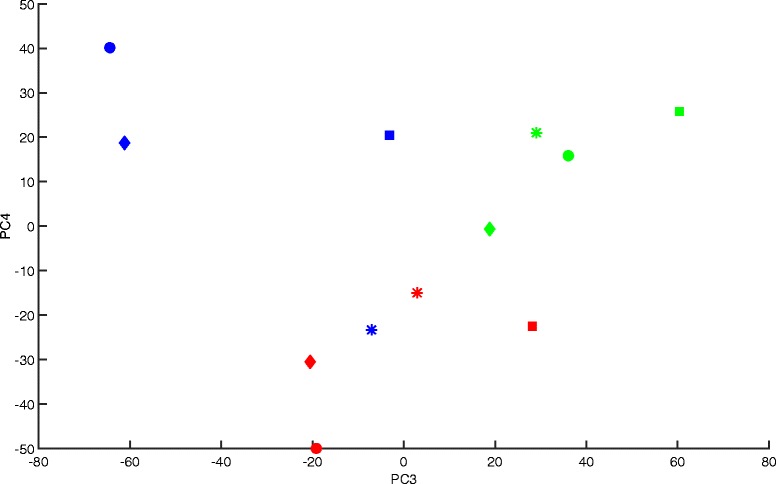
A plot of third and fourth PCs for Dataset 3 (Johnson et al., 2007). The three colours represent the three processing batches. The shapes represent four distinct experimental conditions. The clustering of batches indicates the likely presence of batch effects in the third and fourth PCs of the data

For Datasets 1 and 2 the most of batch related variance is accounted for before the third principal component, which is typically the case given the relative size of batch noise compared to other sources of variation captured in the data. In the case of Dataset 3, there is some correction at the first principal component, none at the second, and the largest correction occurs at the third and fourth principal components. The plot of third and fourth principal components in Fig. 4 shows a clear grouping of scores into processing batches, which suggests that what Harman is identifying and removing as batch noise may indeed be so.

**Fig. 5:**
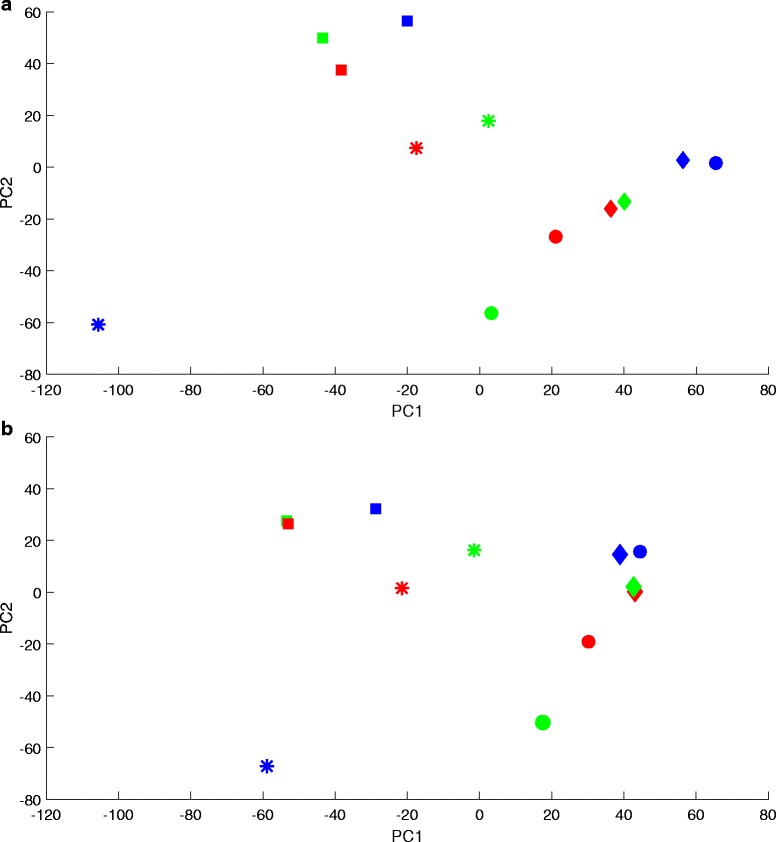
**a** A plot of first and second PCs for Dataset 3 (Johnson et al., 2007) after correction by Harman (.10). The three colours represent the three processing batches. The shapes represent four distinct experimental conditions. **b** A plot of first and second PCs for Dataset 3 (Johnson et al., 2007) after correction by ComBat. The three colours represent the three processing batches. The shapes represent four distinct experimental conditions

Variance in the first two principal components is mainly due to within-treatment variability rather than batch effects (Fig. 3) as mentioned, and yet ComBat removes nearly half of the overall data variance. It may therefore be interesting to see how the first two PCs of the ComBat corrected data look. For a fair comparison, we do the same for Harman at the lowest confidence setting, which maximises the amount of data variance removed. As Fig. 5a shows Harman brings the batches closer to one another by reducing batch means towards zero, but without changing the distribution of samples within them. ComBat, on the other hand, rearranges samples within batch (Fig. 5b), and in particular brings the outlying member of the “*” treatment group within about two thirds of the original distance from the remaining three samples in the batch. More broadly, Fig. 5 displays the compressed nature of samples belonging to the same batch in ComBat corrected data (Fig. 5b) relative to Harman (Fig. 5a). ComBat, in effect, seems to alter and partially remove the biological variance in the data along with removing batch effects. An analysis of variance also confirms this. While both methods drive variance attributable to batch effects to virtually zero (uncorrected data .128; Harman .00018; ComBat .0053), ComBat also removes 23 % of the variance attributable to treatment (uncorrected data .140; Harman .140; ComBat .108), and about 32 % attributable to within treatment variation (uncorrected data .133; Harman .133; ComBat .090). The analyses of Datasets 1 and 2 also show loss of biological variance resulting from ComBat, but to a lesser extent than Dataset 3.

Finally, considering all three datasets, Harman, for a given confidence limit, has a tight range of gPCA *p*-values. For example, for Harman (.95) *p*-values range between .52 and .7 across the three datasets. ComBat varies from .23 to 1.

## Discussion

We developed Harman, first and foremost, to tackle the double edged problem with batch effects – to optimise batch noise removal with the constraint that the risk of also removing genuine biological variance is quantified and kept to a sensible level determined by the user. We evaluated Harman, comparing its performance as a batch noise removal method to that of ComBat. We chose ComBat as the benchmark, as it is overall the best performing one amongst the existing techniques [9, 10]. We used three independent, publically available datasets for this purpose, two of them produced by our laboratory, and the third originally utilised by the developers of ComBat [17].

First of all, gPCA measure we used indicates that Harman and ComBat perform their primary function – they remove batch noise. For all three datasets gPCA *p*-value for batch effect existence increased markedly following batch removal by either method. The confidence limit for Harman does operate as a trade-off coefficient between noise rejection and data variance preservation as expected. As the confidence limit decreased (i.e. tolerance for overcorrection increased), gPCA *p*-value went up and preserved data variance went down.

Second, the data provide compelling evidence that Harman on the whole may be the one with superior performance. At the outset, our expectation was that ComBat would fall somewhere on the curve formed by Harman at different trade-off settings, except that this point may not always be the optimal one for any given application. As it turned out, for Dataset 1 and Dataset 2 ComBat fell below the performance curve of Harman, meaning that there was always a trade-off setting for Harman which results in better noise rejection and better signal preservation at the same time. In the case of Dataset 1, this was true for all trade-off settings. To put it in perspective, Harman with an extremely cavalier confidence limit of .10 (meaning there is 90 % chance that biological signal is being removed along with batch noise) not only displayed better noise rejection, but preserved more data variance than ComBat (see Fig. 1). At the conservative extreme, Harman (.99) which stops removing variance if there is just 1 % chance that it might also be removing genuine signal achieved better noise suppression (gPCA *p*-value = .42) than ComBat while preserving 15 percentage points more data variance. At the typical trade-off setting of .95, the value used in the actual studies [11, 12], Harman returned 63 % data variance with a gPCA *p*-value of .63 against ComBat’s 52 % and with a lower gPCA *p*-value of .23 for Dataset 1. For Dataset 2, Harman (.95) returned 93 % data variance to ComBat’s 79 %, and had a higher gPCA *p*-value (.72 vs .58).

Its peculiarities notwithstanding, Dataset 3 also provides evidence that Harman’s performance may be superior. The gPCA *p*-value for the raw data was .225, significantly larger than those of Dataset 1 and Dataset 2. Interpreting this result as there not being a batch effect in Dataset 3 is the worst possible scenario for both methods. It means that whatever the methods removed from the dataset was biological signal, not batch noise. Combat preserved less data variance than Harman for all confidence limit settings. Harman (.75) matched ComBat’s gPCA *p*-value of 1 yet preserved 20 percentage points more data variance. The difference between Harman (.95) and Combat was a sizable 31 percentage points.

Fortunately for the two batch correction methods, and in particular Harman, further exploration revealed that there may have been a batch noise component in Dataset 3. Harman had identified that the noise component in Dataset 3 was predominantly in the third and fourth principal components. A plot of the two principal components (Fig. 4) showed clearly that samples cluster according to which batch they belong, providing at least subjective evidence that there was a batch noise component. It is unusual for third and fourth principal components to account for more batch noise than the first and second. As a general rule, and as a consequence of batch effects being typically the greatest source of variation in genomic datasets, the earlier the principal component the greater the proportion of batch noise explained. Datasets 1 and 2 constitute typical examples of batch effects, in that first and second principal components account for the bulk of that data’s batch noise component.

This raises another pertinent point. It has been argued that PCA based batch correction approaches do not work well if batch effects are not the greatest source of variation [21, 23]. As exemplified by Dataset 3, Harman investigates all principal components for batch effects, and is able to identify and remove them no matter what their relative size compared to other sources of variation.

A further exploration of Dataset 3 (Fig. 5) revealed that ComBat removed biological variance from the data in the process of removing batch effects. A visual comparison of Fig. 5a and b reveals the within-batch compression ComBat causes. An analysis of variance confirmed that Harman, in distinction to Combat, removed only the variance attributable to batch effects without altering the biological (i.e between treatment and within-treatment) variance. Removing treatment variance leads to an expected increase in false negatives in comparison tests, and removing within-treatment variance leads to an expected increase in false positives. We should also note that analysis of variance attributes all that is attributable to batch effects. This still makes analysis of variance a revealing metric to compare the two methods, when they are set to remove the entirety of the batch effect as identified by it. In the general case, however, it does not replace a metric like gPCA, which is also sensitive to the underlying likelihood of any variance attributed to batch effects.

The final point we will discuss is Harman’s consistency in achieving comparable noise suppression - signal preservation trade-offs across different datasets, which is of particular advantage when conducting meta-analyses and genomic data integration from several distinct datasets [10]. It would be possible to falsely infer differences between two equivalent datasets, just by being bullish in the removal of batch effects in one, and overly cautious in the other. The three datasets varied in the relative magnitude (Dataset 1 vs Dataset 2) and also nature (Dataset 3 vs Datasets 1 and 2) of their batch noise components. They also varied in the number and size of their processing batches. Yet, after correction by Harman (.95), the resulting datasets had a tight range of gPCA *p*-values, from 0.52 to 0.7. This is not accidental. What Harman removes as batch noise is driven directly by a trade-off coefficient constraining it to approach, but not exceed, a set risk of overcorrection. Furthermore this risk calculation is internally normalised for different batch numbers and sizes (see methods section). ComBat on the other hand, resulted in a relatively wide range of gPCA *p*-values, from 0.23 to 1. This difference in consistency between the two methods is similarly reflected in resultant preserved data variance post correction as a function of the level of batch noise in the raw data. Dataset 1 had a much more prevalent batch noise component than Dataset 3. Accordingly Harman (.95) removed 37 % variance from Dataset 1 and 17 % from Dataset 3, settling for comparable gPCA *p*-value scores (.63 and .52, respectively). Combat, on the other hand removed 48 % from Dataset 1, and yet 49 % from Dataset 3, producing quite different gPCA *p*-value scores (.23 and 1, respectively) in the process.

## Conclusion

Considering the issue of batch noise in its totality – the potential impact of its presence (or undercorrection) as well as overcorrection, and the importance of being able to control the trade-off between batch noise rejection and signal preservation especially in relation to studies that span multiple datasets – it is reasonable to state that Harman’s performance as explored in this study makes it the more effective approach to deal with batch effects in high-throughput genomic datasets. Harman is flexible in terms of the data types it can process (e.g. microarray, RNA-seq, methylation). Given its mathematical underpinnings its potential use extends beyond genomic datasets. Of practical significance, it is also able to work with datasets where batch compositions – i.e. the number of experimental conditions, and replicates they contain – are not necessarily the same. It is freely available online as an R package, as well as a compiled Matlab package which does not require a Matlab license to run.

## Methods

### The datasets

In the olfactory stem cell study (Dataset 1), there were six treatment groups plus the control group, each consisting of four replicates, giving a total number of 28 arrays [11]. The experiment was performed with four processing batches of seven arrays each, consisting of one replicate from each of the groups. The dataset comprising the genome wide gene expression scores from the 24 Affymetrix Human Gene 1.0 ST arrays, were normalised and background adjusted as a whole using the RMA procedure [8] in MATLAB. Batch correction methods, ComBat and Harman were performed on the RMA adjusted dataset.

The mouse study (Dataset 2) had four groups (three treatment, one control) with six replicates in each group, making a total of 24 arrays [12]. There were a total of three processing batches of eight arrays, each consisting of two replicates per group. Affymetrix Mouse Gene 1.0 ST arrays were used in this study. The third dataset is the one used by Johnson et al. ([17], p.119). This was another cell study with one treatment, one control, and 2 time points, resulting in 4 distinct (2 treatment x 2 time points) experimental conditions. There were three batches and a total of 12 samples, with each batch consisting of one replicate from each of the experimental conditions. RMA was implemented in the same way as Dataset 1 described above, and batch corrections were applied to RMA adjusted data.

### PCA is an effective means of complexity reduction and data visualisation

Principal component analysis (PCA) is one of the most widely used techniques of multivariate analysis [24]. It is an intuitive way of reducing complexity without any (involuntary) loss of information. A typical gene-expression dataset will have n samples of p (highly inter-related) probesets, where n is typically in the lower range of 10–100, and p is 20,000–40,000. PCA transforms the data into a new set of variables, where n samples are expressed in (n-1) dimensions, and sometimes fewer depending on how extensively inter-related the probesets may be. The new dimensions are the principal components (PCs), which are orthogonal (uncorrelated) to one another, and are ordered according to how much of the data variance they explain. First PC accounts for the largest portion of variance, the second PC accounts for the second most, and the last PC accounts for the least (non-zero) portion of variance. Collectively, principal components account for all of the variance in the data, and as such there is no loss of information. It is also useful to note that principal components are weighted linear sums of the original variables (e.g. probesets) in which the data is expressed.

PCA is routinely used as a visualisation tool for high-throughput genomics data. It is not viable to visualise a particular sample in a 20,000-dimensional probeset space. A two-dimensional plot of first and second PCs, on the other hand provides meaningful, intelligible information while still representing a significant portion of the variance in the data. Indeed, a table of paired plots of many (if not all) PC’s can be produced, which spans virtually all the variance in the data (e.g. [21], p.109, Fig. 5; “PCplot” function in [25]).

### In PC plots batch effects appear as marked differences in batch means

Plots of (the major) principal components are also a very popular means of displaying batch effects. Batch effects, as captured in a given principal component appear as a shift or offset in the geometric centre of the sample scores which belong to the same batch (see Figs. 1b, 2b and 4; see also [15, 16]). This is not incidental. We can assume, for batch effects, the general model of additive as well as multiplicative noise at the measurement (e.g. probe) level [10]. Such measurements are typically log transformed meaning that the resulting noise component is additive only. Moreover, even in the absence of log transformation, as principal components are weighted linear sums of the measurement variables, resulting effect of batch noise will be additive at the level of principal component scores. Because batch effects are by definition common to all samples in a processing batch, they share this additive noise in their PC scores, resulting in an offset in the mean of the batch. Furthermore, what puts these noise related offsets in batch means in sharp contrast is that for a given principal component the sample scores have a mean of zero. Therefore, if not for the batch effects, samples from different (but similarly constituted) processing batches would be statistically equivalent and hence the expected value of batch means would also be zero. In principle, therefore, the more distinct the dispersion of batches, the larger the batch noise component in the dataset.

### From subjective visualisation to quantification of batch noise

**Fig. 6:**
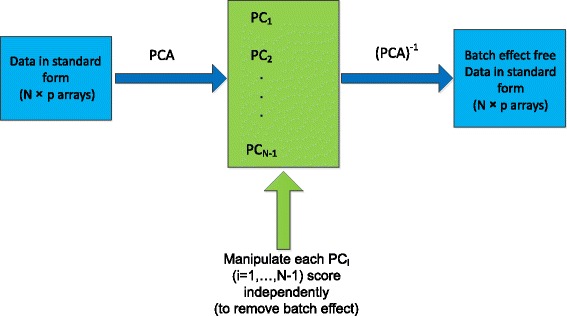
Diagram demonstrates how a potential batch correction procedure may be implemented at each PC. The corrected data can then be transformed from principal components back to the original measurement variables as dimensions. As the dimensionality of the corrected data is identical to the uncorrected original, all downstream analysis of can proceed without requiring any algorithmic adjustments

Capture of batch effects as shifts in batch means in a PC coordinate system forms the basis of an objective assessment of batch effect where batch noise is quantified and then potentially removed. If a correction procedure can be established in the PC coordinate system, all that remains is straight forward matrix algebra to transform the (corrected) samples back into the standard data format, as depicted in Fig. 6 (see also [15, 16, 26]).

The distinct batch noise variance to be found in each of the PCs can be removed independently, which results in the modification of the corresponding column vector of the PC scores matrix. Once all the PCs are corrected, the modified PC scores matrix is transformed back into the original set of variables, i.e. probesets for our Datasets (1-3). The corrected data would consist of the N samples expressed in p probesets as in the original dataset, except that the batch noise component in probeset values is removed. Eq.1 describes this process, assuming that a *correction* procedure exists.

1 $$ \begin{array}{c}\left[ Coef{f}_{p\times \left(N-1\right)}, Score{s}_{N\times \left(N-1\right)}\right]=pca\left(dat{a}_{N\times p}\right)\\ {} Correctedscores\left(:,k\right)= Correction\left( Scores\left(:,k\right)\right)\kern1.5em for\kern1em k=1,\dots, N-1\\ {} Correcteddat{a}_{N\times p}=pc{a}^{-1}\left( Correctedscore{s}_{N\times \left(N-1\right)}\right)= Correctedscore{s}_{N\times \left(N-1\right)}* Coef{f}_{\left(N-1\right)\times p}^{\prime}\end{array} $$

It should be mentioned that the probeset means are subtracted from the data prior to *pca*, and then added to the *Correcteddata* after *pca*^*−1*^. As denoted above, *pca*^*−1*^ amounts to a matrix multiplication (by the transpose of the *coeff* matrix computed by PCA*)* and the resultant *Correcteddata* is unique.

The key issue to consider in terms of establishing a correction procedure is the converse of what is described in the previous sub-section. If batch noise to be found in a given principal component is necessarily and exhaustively reflected as shifts in the mean scores of individual batches, can such shifts observed in PC scores be wholly and directly be attributed to batch effects? If there were no batch effects, the expected mean of each batch would be zero because the overall mean of PC scores is zero. And if there were hundreds of samples in a batch, we would expect the actual mean of the batch to be very close to the expected mean. In which case, a satisfactory batch effect correction procedure may amount to no more than removing the batch mean from the scores that constitute that batch. Typically, though, the number of samples in a batch is relatively small. The batch sizes of the datasets we analysed in this study, for example, varied between 4 and 8. We would thus expect that the actual batch means would vary considerably around the expected mean of zero. As such, we would not be able to say without further investigation, whether a particular non-zero batch-mean is a reflection of the existence of batch effects, or whether it is a reasonable variation between the “population mean” (of zero) and that of a small subset from that population. Essentially, the way Harman identifies whether or not batch effects exist in a given principal component of the dataset is by calculating the overall likelihood of the observed deviation of batch means from zero, as a function of the size of batches and the total number of samples.

It may be helpful to look in detail at how this batch noise quantification works, using Dataset 1 to illustrate the process. There are four batches in this dataset, each possessing one of the four replicates from seven treatments (see Fig. 1b). What would it mean to assume that there are no batch effects in this data? It would necessarily follow that there is statistically speaking no difference between, say, the square in the cyan batch and the squares in the red, green and blue batches – we have assumed after all that there is no batch specific component to the PC score denoted by the cyan square, or any of the other squares. The difference between four squares would then reflect the variability of the treatment of which they are replicate PC scores. It would also mean that the cyan square in the cyan batch happens to be there by chance. Any of the four squares (i.e. 4 PC scores belonging to that treatment) could have belonged to the cyan batch. This would be true for all of the treatments and their replicates.

If this is so, then the four batches can be seen as having eventuated from a much larger population of potential batches. Since there are seven treatments in a batch, and each treatment has four replicates, then there are 4^7^ possible combinations of PC scores each constituting a potential batch. For the general case, number of possible combinations is:

$$ {\displaystyle \prod_{\alpha =1}^{\tau}\left(\begin{array}{c}\hfill {n}_{\alpha}\hfill \\ {}\hfill {k}_{\alpha}\hfill \end{array}\right)} $$, where

*τ* = number of distinct treatments in a batch,

n_α_ = total number of replicates of treatment α in the study,

k_α_ = number of replicates of treatment α in batch.

By computing the mean of the potential batches, we can establish the population distribution of batch-means representing the no-batch-effect assumption. We can use this distribution to calculate the empirical likelihood of ending up with the four actual batch-means under the assumption that there are no batch effects.

The batch-mean population is normally distributed, irrespective of the distribution of measurement variables (e.g. probesets) in the raw dataset. This is because of Central Limit Theorem [27], which applies not once but twice. Central Limit Theorem states that populations created from sums or averages of large numbers are normally distributed (asymptotically speaking) irrespective of the underlying distribution of those numbers. PC scores are weighted linear sums of the original measurement variables (i.e. probes), which number in the thousands in typical high throughput datasets, and in the ones we use in this study. Batch-means in turn are weighted linear sums of PC scores. We would also expect the mean of this distribution to be zero, on account of the PC scores adding up to zero. The critical measure derived from the establishment of the population distribution of batch-means is its variance (or standard distribution). Once the batch-mean population is established, it is trivial to compute its variance.

After establishing the population distribution of batch-means – most crucially, its variance – representing the condition that there are no batch effects, we proceed to calculating the probability (z_*b*_) of selecting a batch *b* with a particular batch-mean (BM_*b*_). Each batch mean probability is calculated based on the cumulative distribution function (CDF) of the population distribution [28].

2 $$ \begin{array}{c}F(x)=CDF\left( normal,0,std,x\right)\\ {}{z}_b= probability\left(B{M}_b\right)=F\left(-\left|B{M}_b\right|\right)\end{array} $$

We negate the absolute value of BM_*b*_ in the formula, as the probability of deviating from the expected batch mean is a function only of the magnitude of the deviation, not its direction. The overall probability (*L*) of the four actual batch-means eventuating, will be a function of the probability of the individual batch-means, with the constraint that they must add up to zero. If there were no constraining equation, L would be the product of the individual batch-mean probabilities. Note that with this constraint, once the three batch-means are chosen, the fourth one is fixed. There are four distinct ways of choosing a set of three batch means in this way. Hence the structure of L becomes:

$$ L=f\left({z}_1,{z}_2,{z}_3,{z}_4\right)\kern1em with\kern1.5em {\displaystyle \sum_{b=1}^4B{M}_b}=0\kern0ex L=c\left({z}_1{z}_2{z}_3+{z}_1{z}_2{z}_4+{z}_1{z}_3{z}_4+{z}_2{z}_3{z}_4\right) $$

where *c* is the normalising constant.

The normalising constant in the equation above plays an important role. First and foremost, we would want *L* to be comparable across different datasets which may have different number of batches. As it stands, *L* is a function of the number of batches in the dataset. Secondly, we would want *L* to range from 0 to 1. The maximum value *L* can have (*L*_*max*_*)* in the example above, is when all batch means are equal to the expected mean of zero. In which case, z_i_ = 0.5 for all values of i, with *L*_*max*_ = *c*(4/8). With *c* thus set to (8/4) to make *L*_*max*_ equal to 1,

$$ L=\frac{8}{4}\left({z}_1{z}_2{z}_3+{z}_1{z}_2{z}_4+{z}_1{z}_3{z}_4+{z}_2{z}_3{z}_4\right) $$

The general equation for n batches is:

3 $$ \begin{array}{l}L=\frac{2^{n-1}}{n}{\displaystyle \sum_{i=1}^n\left({\displaystyle \prod_{j=1}^n{z}_{ij}}\right)\kern2em \mathrm{where}}\kern1em {z}_{ij}={z}_j\kern1.5em  if\kern1em i\ne j\\ {}\kern15em {z}_{ij}=1\kern2em  if\kern1.5em i=j\end{array} $$

With *L*, we now have the likelihood of batch-mean dispersion we observe in the PC scores (normalised with respect to no dispersion, i.e. zero batch-means) if there were no batch effects. If *L* is small, we can say with (1-*L*) confidence that there is batch noise in the data.

### Removal of batch noise

Say the confidence percentage is high – i.e. higher than the smallest value (i.e. confidence limit) at which the user is prepared to say that there are batch effects in the data. This would mean that a portion of the batch-mean dispersion is due to there being batch noise. For a given PC, the scores for the samples can be expressed as:

4 $$ {s}_{ji}=B{M}_j+{r}_{ji}\kern0.5em ,\kern0.5em i=1:n\kern0.5em \mathrm{and}\ \mathrm{j}=1:\mathrm{b}, $$

where *s*_*ij*_ is the score corresponding to i^th^ sample in batch j with batch-mean *BM*_*j*_, n is the number of samples per batch, and b is the number of batches. *r*_*ji*_ thus becomes the distance between the sample score *s*_*ji*_ and centre of the batch to which it belongs.

Removing batch noise would then amount to ‘compressing’ or ‘shrinking’ the observed batch mean dispersion as much as possible, with the constraint that the confidence value is not less than the limit set by the user. In other words, the corrected version of s_ji_ can be defined as:

5 $$ {s}_{ji}(corrected)=k.B{M}_j+{r}_{ji},\kern0.5em 0\le \mathrm{k}<1,\ \mathrm{such}\ \mathrm{that}\ \mathrm{L}\left({z}_j(corrected)\right)=1- confidencelimit $$

In practice, a sufficiently close approximation $\left(\widehat{k}\right)$ to k can be computed iteratively, starting from 1 and approaching zero in discrete steps (e.g. of .01), recomputing *L* at each step and then choosing the smallest $\widehat{k}$, such that the confidence percentage is not less than the confidence limit. Harman uses an optimised version of this process to ensure that the number of iterations is minimised for computational efficiency. For example, suppose the resulting *L* for a given principal component of the data was only .01, meaning that the observed dispersion of batch means only had 1 % chance of emerging in the absence of any batch noise in the data. The user may have decided that a suitable noise rejection – signal preservation trade-off would result from a confidence limit of .95. The corrected scores would be calculated in accordance with the equation above, by compressing batch means with a suitable $\widehat{k}$, such that *L* = .05. This process is repeated independently for all of the PCs.

**Fig. 7:**
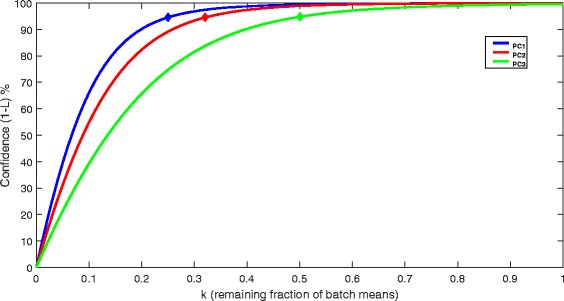
The plot above demonstrates how the confidence (%) for there being no batch effects reduces as the batch means are compressed proportionately towards zero. The three curves correspond to the first three principal components of Dataset 1. For each point on a given curve, batch means are multiplied by the corresponding value of *k* for the purposes of computing the confidence value. The diamond markers on the curves denote the k values (i.e. the resulting batch mean compression) for a chosen confidence limit of 95 %

Figure 7 demonstrates the confidence percentage as a function of k for the first three PCs. The points marked on the three curves correspond to Harman (.95), showing the k values which result from setting the confidence limit to 95 %.

**Fig. 8:**
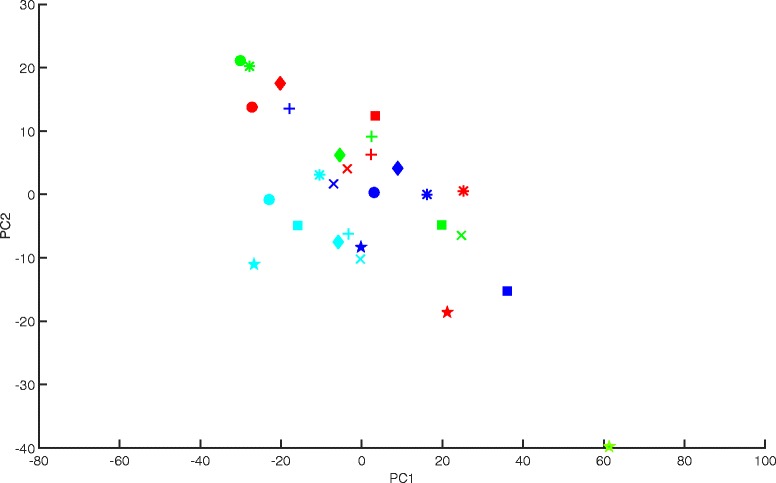
First and second PCs for Dataset 1 (Osmond-McLeod, Osmond et al., 2013) after correction by Harman with a confidence limit of .95. The four colours represent the four processing batches. The shapes represent seven distinct treatments. The clustering of batches before correction (Fig. 1b) indicate the presence of batch effects in the first and second PCs of the data. After correction, the batch means are reduced to .26 (PC1) and .33 (PC2) of their original values

Figure 8 shows the sample scores for the first and second PCs after correction by Harman (.95). The correction vector, i.e. values of k corresponding to all PCs are included in Table 2.

Once all the PCs are corrected, the batch noise free data is expressed in the original variables, as described by Eq.1.

## Additional file

Additional file 1: Contains additional information and discussion on gPCA (Reese et al., 2013). **Table S1.** Demonstrates the inverse proportionality between gPCA *p*-value and the associated ‘delta’ score, reflecting unadjusted relative magnitude of batch effects (Reese et al., 2013). The table shows the scores for all three datasets. **Figure S1.** Contains an Illustration to further help interpret gPCA *p*-value vs preserved data variance plots. (DOCX 60 kb)

## Abbreviations

PC: Principal component
PCA: Principal component analysis
gPCA: Guided-PCA
RNA-seq: RNA sequencing
