# Supplementary material for: Risk-conscious correction of batch effects: maximising information extraction from high-throughput genomic datasets
Source: BMC Bioinformatics. 2016 Sep 1;17(1):332. doi: 10.1186/s12859-016-1212-5 (PMC5009651; doi:10.1186/s12859-016-1212-5)
Supplement: Additional file 1: — Contains additional information and discussion on gPCA (Reese et al., 2013). Table S1. Demonstrates the inverse proportionality between gPCA p-value and the associated ‘delta’ score, reflecting unadjusted relative magnitude of batch effects (Reese et al., 2013). The table shows the scores for all three datasets. Figure S1. Contains an Illustration to further help interpret gPCA p-value vs preserved data variance plots. (DOCX 60 kb) [file 12859_2016_1212_MOESM1_ESM.docx]

## Performance plots

Fig.s1 below describes the features of the two-dimensional plots we use to depict the relative performance of the batch removal techniques. The points on the plot are computed from ComBat and Harman corrected datasets. Y-axis shows the p-value of the guided-PCA statistic developed by Reese and colleagues (2013). The larger the p-value the less likely the probability of existence of batch effects in the corrected dataset. X-axis is the proportion of raw data variance that is preserved after applying the batch correction techniques. An x-value of .8 for example indicates that the batch correction process has removed 20% of the variance of the original dataset. It is worth noting that the X-axis reflects how much data variance – which is a combination of signal and noise components – has been preserved. Nevertheless, when combined with the gPCA p-value it does reflect a relative measure of signal preservation between two correction methods. For example, in Fig.s1, ‘b’ has better signal preservation than ‘a’, since they both have the same gPCA p-value yet b preserves more data variance than a. The extra variance does not contribute to an increased probability of batch noise presence, therefore for it must stem from the signal component. ‘c’ on the other hand, has better noise rejection than ‘b’, and better noise rejection as well as signal preservation than ‘a’.

As stated previously, Harman has an adjustable confidence limit which enables the user to control the risk of overcorrection – that is, removing biological signal along with batch noise. A confidence limit of .90 means that there is a 10% chance what is being removed is not just batch noise but also contains genuine biological signal. Normally, it would not be wise to use too small (.e.g. .10) or too large (.99) a confidence limit. We, however, use an entire spectrum of values here for evaluation purposes. First, the confidence limit is essentially a trade-off coefficient between noise rejection and signal preservation, therefore we would expect to see this reflected in the gPCA p-value vs preserved data variance plots. As we reduce the confidence limit, proportion of preserved variance should decrease, while gPCA p-value increases. This means that there is a performance curve rather than a single point for Harman, and the user can decide where they would like to be on that curve by controlling the confidence limit. Second, this may enable a fairer and more informative comparison between ComBat and Harman. Indeed, our expectation prior to conducting the analysis was that ComBat would most probably fall somewhere on the Harman curve, except that as ComBat may not have a direct adjustment for the noise rejection- signal preservation trade-off, this point on the curve may not always be the optimal one for any given application. Should ComBat fall below the Harman curve, this would mean that it is inferior to Harman in a more fundamental way – i.e. that Harman can be tuned to outperform ComBat on noise rejection and signal preservation at the same time. Similarly, if ComBat happens to fall above the curve, then it could in principle be deemed superior to Harman.

## Guided PCA

Since we use gPCA p-value statistic (Reese et al., 2013, p.2) to measure the efficacy with which ComBat and our novel method removes batch effects, a brief introduction and discussion may be useful. This is a simplified version, limited to only those aspects of gPCA which we use. Essentially, the difference between conventional principal component analysis and gPCA is a sensible and useful compression of the dataset from individual samples to batches, upon which PCA is performed. If we have a genomic data matrix X(s,p) of s samples of p genomic features (e.g. s microarrays with p probesets each), gPCA performs principal components analysis not on X(s,p) but on gX(b,psum), where b denotes the number of batches, and each genomic feature, psum is calculated by adding the corresponding probeset scores of arrays in that batch.

This compression, in effect, aligns the variance of gX with batch rather than treatment differences. After principal components analysis is performed on gX(b,psum), the variance of the first principal component is computed. This score is then compared to the scores of a thousand other pseudo-batches, which are formed by randomly combining samples from X(s,p). If we do have a real batch effect, we would expect the variance of the first principle component of gX(b,p) to be much larger than that of a randomly generated pseudo-batch. P-value is estimated by the proportion of pseudo-batches with a variance larger than that of the actual batch. The gPCA p-value is inversely proportional to the magnitude of the batch effect as calculated by gPCA (Reese et al., 2013). Table S1 below illustrates this for the three datasets used in this study. We used the publically available R package provided by Reese to compute gPCA pvalues (<http://cran.r-project.org/web/packages/gPCA/index.html>).

| Dataset 1 | gPCA delta (batch effect magnitude ) | gPCA p_value | Dataset 2 | gPCA delta (batch effect magnitude) | gPCA  p_value | Dataset 3 | gPCA delta (batch effect magnitude) | gPCA  p_value |
| --- | --- | --- | --- | --- | --- | --- | --- | --- |
|  |  |  |  |  |  |  |  |  |
|  |  |  |  |  |  |  |  |  |
| Raw Data | 0.9475394 | 0.008 | Raw Data | 0.805056 | 0.037 | Raw Data | 0.756648 | 0.225 |
| ComBat | 0.7542261 | 0.233 | ComBat | 0.4908377 | 0.571 | ComBat | 0.06634689 | 1 |
| Harman (.99) | 0.6887331 | 0.42 | Harman(.99) | 0.615 | 0.282 | Harman (.99) | 0.7566478 | 0.224 |
| Harman (.98) | 0.6198316 | 0.548 | Harman (.98) | 0.522452 | 0.49 | Harman (.98) | 0.7330958 | 0.295 |
| Harman (.97) | 0.5822056 | 0.588 | Harman (.97) | 0.476045 | 0.569 | Harman (.97) | 0.7454518 | 0.284 |
| Harman (.95) | 0.5394578 | 0.633 | Harman (.95) | 0.425657 | 0.715 | Harman (.95) | 0.6292926 | 0.515 |
| Harman (.90) | 0.483996 | 0.712 | Harman (.90) | 0.386793 | 0.793 | Harman (.90) | 0.4272403 | 0.897 |
| Harman (.85) | 0.4475337 | 0.765 | Harman (.85) | 0.35586 | 0.849 | Harman (.85) | 0.3417458 | 0.968 |
| Harman (.75) | 0.3863463 | 0.83 | Harman (.75) | 0.27433 | 0.947 | Harman (.75) | 0.2142215 | 0.997 |
| Harman (.65) | 0.3244486 | 0.874 | Harman (.65) | 0.217504 | 0.98 | Harman (.65) | 0.1719732 | 1 |
| Harman (50) | 0.3031469 | 0.888 | Harman (50) | 0.1898185 | 0.993 | Harman (50) | 0.1445171 | 1 |
| Harman (.40) | 0.2338068 | 0.924 | Harman (.40) | 0.1807688 | 0.997 | Harman (.40) | 0.1294362 | 1 |
| Harman (.30) | 0.3267529 | 0.88 | Harman (.30) | 0.1646077 | 0.997 | Harman (.30) | 0.1174246 | 1 |
| Harman (.10) | 0.1129104 | 0.991 | Harman (.10) | 0.1370826 | 1 | Harman (.10) | 0.08611695 | 1 |

TABLE S1: Demonstrates the inverse proportionality between gPCA p-value and the associated ‘delta’ score, reflecting unadjusted relative magnitude of batch effects (Reese et al., 2013). The table shows the scores for all three datasets.

## Figure Caption

Fig.s1: An illustration of the interpretation of gPCA p-value vs preserved data variance plots. Points *a*, *b*, and *c* represent to the same dataset corrected in three different ways. *a* and *b* have equivalent noise components, while *b* preserves more data variance. This means that *b* is able to match *a*’s noise removal, with a smaller data variance loss. *c* and *b* preserve the same amount of data variance, yet in the case of *c*, this variance is less likely to be due to noise. Therefore *c* removes more noise than *b* for the same data variance cost. *c* thus removes more noise than *a* and for a smaller data variance cost. *c* represents the most effective method, followed by *b*, with *a* being the least effective.

## Table Caption

TABLE S1: Demonstrates the inverse proportionality between gPCA p-value and the associated ‘delta’ score, reflecting unadjusted relative magnitude of batch effects (Reese et al., 2013). The table shows the scores for all three datasets.
